# Executive Dysfunction—A Serious Game for Assessment in Real-Life Contexts: Development and Cross-Sectional Usability Study

**DOI:** 10.2196/94860

**Published:** 2026-08-27

**Authors:** Daniela Olivares, David Martínez-Pernía, Loreto Olavarría, Mauricio Cerda, Diego Montenegro, Cristian Ramírez, Rodrigo Nieto, Gonzalo Farías, Andrea Slachevsky

**Affiliations:** 1 Neuropsychology and Clinical Neuroscience Laboratory (LANNEC), Interdisciplinary Nucleus for Physiology, Biophysics and Pathophysiology– Institute of Biomedical Science, Neuroscience and East Neuroscience Departments, Faculty of Medicine, University of Chile, Santiago, Santiago Metropolitan, Chile; 2 Doctoral Program in Neuroscience, School of Medicine, Pontificia Universidad Católica de Chile, Santiago, Chile; 3 Center for Social and Cognitive Neuroscience (CSCN), School of Psychology, Adolfo Ibáñez University, Santiago, Chile; 4 Geroscience Center for Brain Health and Metabolism (GERO), Santiago, Santiago Metropolitan, Chile; 5 Department of Computer Science, Faculty of Physical and Mathematical Sciences, University of Chile, Santiago, Chile; 6 Neuroepigenetic Regulation Lab, Faculty of Medicine, Pontificia Universidad Católica de Chile, Santiago, Chile; 7 Department of Psychiatry and Mental Health, Faculty of Medicine, University of Chile, Santiago, Chile; 8 Neuroscience Department, Faculty of Medicine, University of Chile, Santiago, Chile; 9 University Psychiatric Clinic, Clinical Hospital of the University of Chile, University of Chile, Santiago, Chile; 10 Center for Advanced Clinical Research (CICA), Hospital Clínico de la Universidad de Chile, Santiago, Chile; 11 Department of Neurology North, Faculty of Medicine, University of Chile, Santiago, Chile; 12 Memory and Neuropsychiatric Center (CMYN) Neurology Department, Hospital del Salvador & Faculty of Medicine, University of Chile, Avenida Salvador 486, Santiago, 7500922, Chile, 56 2 2978 6148; 13 Neurology and Psychiatry Department, Clínica Alemana-University Desarrollo, Santiago, Chile

**Keywords:** executive dysfunction, ecological validity, serious games, social cognition, contextual control, usability, traumatic brain injury, TBI, COVID-19

## Abstract

**Background:**

Traditional neuropsychological assessments often lack ecological validity and have limited sensitivity for detecting executive dysfunction (ED) in daily life. Although grounded in standardized tasks, they do not represent the complexity of real-world contexts. Serious games (SGs) have emerged as a promising approach by simulating ecologically valid environments involving dynamic, emotional, and socially embedded interactions.

**Objective:**

This study aimed to (1) describe the development of a clinically oriented SG for assessing executive functioning in ecologically valid scenarios, and (2) evaluate its usability in clinical and healthy populations.

**Methods:**

We conducted a cross-sectional usability study using a theory-driven SG set in a restaurant environment, developed through an iterative process based on neuropsychological theory and expert input. Participants assumed the role of a waiter and completed tasks targeting multiple executive domains (eg, planning, working memory, decision-making, contextual control, social cognition, prospective memory, and multitasking), with embedded emotional and social cues. A total of 181 participants (43 with traumatic brain injury [TBI], 49 post–COVID-19, and 89 healthy controls), aged 21 to 66 years, were recruited through convenience sampling from clinical and community settings. ED was classified based on standardized neuropsychological assessment and clinical consensus involving neurologists and neuropsychologists. Usability and perceived difficulty were assessed using the System Usability Scale and Perceived Difficulty Scale. Group differences were analyzed using Kruskal-Wallis tests with post hoc comparisons, and generalized linear and ordinal logistic regression models adjusted for age, sex, and education (α=.05).

**Results:**

A novel SG designed to support the assessment of executive functioning provides a more ecologically valid context than traditional neuropsychological tests. Usability was rated as good across groups, with mean System Usability Scale scores of 73.9 (SD 17.6) in TBI, 76.5 (SD 14.5) in post–COVID-19, and 81.7 (SD 13.5) in controls. Significant differences were observed across groups (*χ*²_2_=9.18, *P*=.01), with lower usability in TBI compared to controls. Participants with ED (n=59) showed lower usability (mean difference −8.6, 95% CI −13.2 to −4.0; *P*<.001) and higher perceived difficulty (mean difference 1.3, 95% CI 0.5‐2.1; *P*<.001), though these differences were no longer significant after adjusting for age and education. Age was associated with lower usability (β=−.27, 95% CI −0.45 to −0.09; *P*=.003) and higher difficulty (odds ratio 1.04, 95% CI 1.02‐1.07; *P*<.001), while higher education was associated with better usability (β=.79, 95% CI 0.04‐1.54; *P*=.04).

**Conclusions:**

This study presents a novel SG integrating emotionally and socially embedded tasks to assess executive functioning in ecologically valid contexts, extending prior work through the inclusion of clinical populations and real-world demands. Findings indicate good usability and underscore the influence of demographic factors. This approach enhances the ecological relevance of neuropsychological assessment and supports its application in clinical and digital health settings. Further research should evaluate diagnostic accuracy, reliability, and real-world implementation.

## Introduction

### Executive Dysfunction and Its Assessment

Executive functions (EFs) arise from the interaction of multiple cognitive domains and are defined as the cognitive abilities necessary for goal setting, efficient execution of actions, and flexible modification of behavior to adapt to changing contexts [1,2]. Executive dysfunction (ED), a disruption of these abilities, is a frequent consequence of brain-related disorders, including traumatic brain injury (TBI), dementia, and schizophrenia and, more recently, has been recognized as a sequel of COVID-19 [3]. ED is a major contributor to psychosocial disability in these patients, affecting their ability to perform cognitively demanding activities such as employment and social participation [4-6]. This loss of functional independence has been directly linked to increased health care costs, with a linear relationship observed between the severity of ED and the economic burden associated with these disorders [7,8].

Early recognition of ED is essential to identify patients’ difficulties and facilitate timely access to social and health services. However, current assessment methods demonstrate limited ecological validity and low sensitivity, often failing to detect executive difficulties that manifest in daily life [9], limiting timely access to necessary health care services and psychosocial support. Although a wide range of traditional methods exist, including paper-and-pencil tests and naturalistic observations, expert clinicians frequently identify ED in patients who perform adequately on standard evaluations [10,11]. There is consensus that traditional clinical assessments have lower sensitivity compared to expert clinical judgment [12]. In fact, standard evaluations fail to detect ED in up to 58% of individuals with TBI [13], likely due to their inability to reflect real-world cognitive demands. This detection gap has been attributed to several factors: (1) conventional tests often do not assess critical domains of executive functioning necessary for everyday life, such as contextual adaptation integrating cognitive and emotional information, prospective memory, and multitasking within social contexts; (2) they rely on highly structured tasks; and (3) they measure performance through a limited number of discrete or categorical variables (eg, correct responses, errors, and overall task completion time) [12,14,15].

### Serious Games for EF Assessment

Information and communication technologies have enabled the development of novel, computer-based tools—notably serious games (SGs)—to enhance the sensitivity and ecological validity of ED assessments. SGs are interactive digital applications designed with purposes beyond entertainment, including assessment, education, or rehabilitation in health contexts [16,17]. SG-based evaluations facilitate the assessment of EF within simulated real-life contexts through unstructured tasks, allowing the inclusion of EF domains that are not typically evaluated [14]. These tools are easy to implement in clinical settings, require only standard, low-cost technology (eg, computers or tablets), are simple for health care personnel to administer, are well tolerated by patients, and are applicable across a broad spectrum of brain disorders. Recent studies have increasingly explored the use of SG for cognitive assessment and rehabilitation, highlighting their potential to enhance ecological validity and user engagement [14,18,19]. However, much of this research has been conducted in nonclinical populations or small samples [20,21]. Although SGs have been proposed as a way to enhance ecological validity by simulating real-world contexts, it is important to keep in consideration that it may also increase cognitive load, which can influence user performance and experience [22,23].

Additionally, performance can be assessed through multiple indicators, including continuous measures that are often overlooked by conventional neuropsychological tests. Furthermore, advanced analytical approaches, such as machine learning models, provide a powerful framework for extracting clinically meaningful insights from these complex, multidimensional data [24].

### Importance of Usability Studies in SGs for Cognitive Assessment

Beyond diagnostic sensitivity, the clinical applicability of SGs also depends on their usability, particularly for individuals with cognitive impairments. Usability can be defined as the degree to which a product can be used effectively, efficiently, and satisfactorily by specific users in a particular context [16,25]. In neuropsychological applications, this includes learnability, ease of navigation, user control, and satisfaction with game features. Poor usability may overload cognitive resources, hinder learning, and reduce motivation to engage with the task [26-28]. Although usability studies have been conducted for SGs [20,25,29,30], most have involved small samples or participants who do not represent the intended clinical population [21,31,32]. Given that SG users may belong to nongaming populations with heterogeneous cognitive profiles, it is critical to assess usability in the specific clinical groups for whom these tools are designed [30]. Although some studies have examined usability aspects, these have often been limited to specific populations or have not incorporated complex, socially and emotionally embedded scenarios that reflect real-world executive demands [21,33]. As a result, there remains a need for clinically oriented SG that combines ecological validity with systematic usability evaluation in populations affected by ED.

### Objectives

The objective of this study was to introduce a new SG developed to assess EFs in adults. The aims of this study were (1) to describe the development of a clinically oriented SG designed to assess ED in ecologically valid contexts and (2) to evaluate its usability in clinical and healthy populations using standardized measures of usability and perceived difficulty.

## Methods

### SG Development

Our team developed a novel computer-based tool for the assessment of EF through an SG. The development process followed an iterative, theory-driven design, incorporating input from expert clinicians and cognitive scientists. Expert judges, comprising national and international neuroscientists, neurologists, and neuropsychologists, participated in a structured validation process. They contributed to the selection of the EF domains to be assessed, the design of the SG’s central metaphor, a restaurant setting in which the player assumes the role of a waiter, and the specification of the tasks designed to evaluate core executive processes. This “waiter scenario” was intentionally chosen to simulate cognitively demanding situations grounded in everyday life, particularly those requiring rapid decision-making, emotional regulation, and cognitive flexibility—capacities often compromised in individuals with ED. Working as a waiter involves managing multiple concurrent events (eg, taking orders, responding to customer feedback, and coordinating with kitchen staff), all within a socially and emotionally dynamic environment. These conditions create an ideal context for embedding emotionally salient and contextually rich cues that reflect the complexity of real-life cognitive demands. Special attention was given to enhancing ecological validity and contextual realism. For example, the game’s graphic design emphasized clearly identifiable facial expressions to support emotional recognition and contextual control during gameplay. Tasks integrate context-dependent problem-solving, emotionally charged stimuli, and simultaneous goal management, components that, according to recent theoretical frameworks [14], are essential for assessing ED in ecologically valid settings.

### Expert Consensus

The SG was designed to operationalize 9 key EF domains, selected based on expert consensus and their relevance to real-life functioning. Table 1 presents each cognitive domain, its conceptual definition, and how it is operationalized within the gameplay. The in-game tasks were crafted to embed these domains in naturalistic interactions, ensuring ecological validity by incorporating emotionally salient cues, simultaneous task demands, and context-rich decision-making scenarios. This structure allows for the assessment of ED as it emerges in realistic, socially embedded environments, rather than through artificial, isolated tasks.

To empirically evaluate the alignment between expert ratings and the SG’s proposed indicators of ED, the Cohen κ coefficient [34-36] was used. This statistic is suitable for assessing interrater agreement in categorical or ordinal data across multiple evaluators. Content validity was supported by high levels of expert agreement, with at least 60%—and in most cases over 80%—of experts rating each proposed domain and corresponding task metric as appropriate for assessing ED.

**Figure 1.**
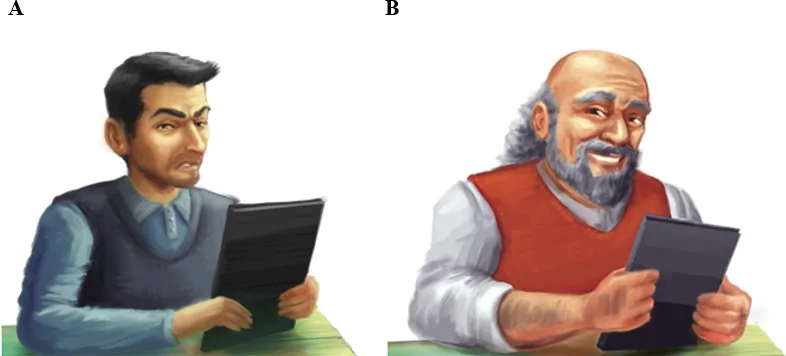
Illustration of customer characters used in the serious game, displaying 2 emotional expressions, (A) Anger and (B) Happiness to simulate socially and emotionally relevant interactions.

Following the review of these domains, experts also evaluated a set of 6 character illustrations designed to portray basic emotional expressions (sadness, disgust, anger, happiness, neutral, and surprise). These visual stimuli were used to assess emotional recognition and the player’s capacity to adapt behavior according to social signals, a core component of contextual control and social cognition. Special care was taken to ensure that facial expressions were visually distinct and ecologically valid, providing reliable cues for real-time emotional appraisal and decision-making. Figure 1 depicts the examples of customer characters expressing anger and happiness.

**Table 1.** Definition of cognitive domains and their operationalization within a clinically oriented serious game designed to assess executive dysfunction.

Cognitive domain	Conceptual definition	Operationalization definition
Time-based prospective memory	Ability to remember to perform a postponed action after a predetermined time interval	Remember to play music every time it is switched off. Indication is given that music should always be playing in the restaurant
Event-based prospective memory	Ability to remember to perform an action in response to an environmental cue	Execution of a task when a predefined condition occurs within the game context
Working memory	Limited-capacity system for temporary storage and manipulation of information	Retention and short-term recall of information required to complete tasks
Planning	Ability to set goals and organize intermediate steps	All actions or strategies performed by the participant to achieve a specific goal
Decision-making	Ability to evaluate potential outcomes of different actions	Choice between alternative actions with distinct positive, neutral, or negative consequences
Cognitive flexibility	Ability to adapt behavior to changing demands	Behavioral adjustment in response to novel or changing game conditions
Contextual control	Ability to regulate behavior based on contextual emotional or nonverbal cues	Adaptation of responses to customers’ petitions or facial expressions
Social cognition	Ability to understand others’ intentions and social norms	Conventional behavior aligned with social expectations during interactions
Multitasking	Ability to manage multiple tasks simultaneously or sequentially	Concurrent or alternating the execution of multiple in-game tasks

### Development of the SG

#### Game Tracking

The SG was implemented on a standard laptop using a mouse for navigation. All player interactions were dynamically tracked through an integrated recording system, which captured performance metrics throughout the restaurant environment (including tables, kitchen, and service zones). Each action was logged along with contextual parameters such as the emotional state of customers, concurrent tasks, and environmental demands. States were defined by sequences of actions, with metrics such as execution times, decision choices, errors, and the simultaneous state of all tables recorded in real time. This dynamic recording generated a substantial volume of data, organized into a JSON file structure. This format enabled both macrolevel and microlevel analyses, including reaction times, error rates, and the reconstruction of player behavior across the full temporal sequence of gameplay. In the present publication, we focus on the development of the SG and its usability evaluation; performance data and gameplay metrics involving both healthy and clinical populations will be presented in future studies.

#### Gameplay Procedure and Structure

**Figure 2.**
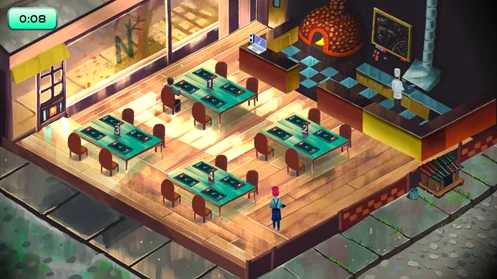
Serious game environment representing a restaurant setting used to simulate ecologically valid scenarios for the assessment of executive functioning.

The SG is intended to be administered in the context of a neuropsychological evaluation, under the supervision of a health professional. The game’s setting is situated within the context of a restaurant, wherein the player assumes the role of a waiter (Figure 2 for the SG environment).

**Figure 3.**
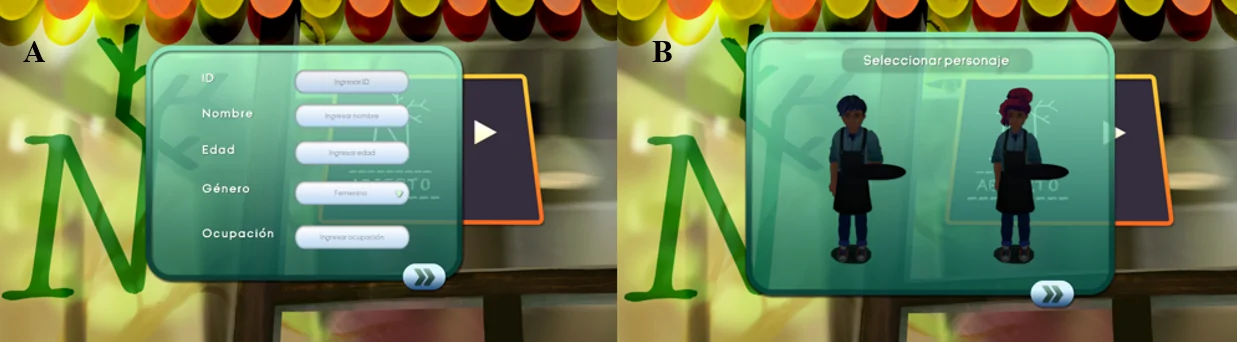
(A) Home screen and (B) avatar selection interface of the serious game, allowing users to choose their in-game character before gameplay.

Initially, participants are required to provide their full name (which is used to personalize interactions with customers during the game), age, and sex (female, male, or other). The following step involves the selection of an avatar from a pair of possible options. Figure 3 shows the home screen and avatar selection.

**Figure 4.**
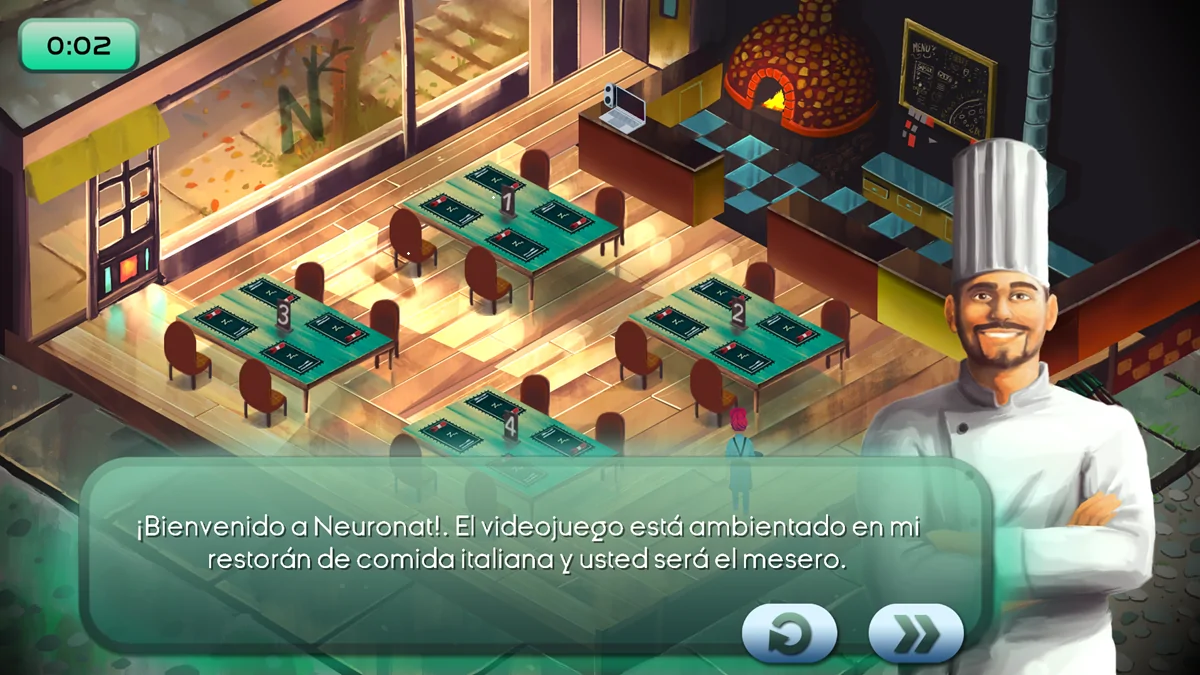
Initial tutorial of the serious game. In this phase, players are introduced to the game mechanics and instructions.

The game begins with a tutorial phase in which a character (the chef) gives instructions and shows the steps necessary to achieve the proposed objectives (see Figure 4). The purpose of this phase is to familiarize the person with the virtual environment and the tasks to be performed when the evaluation begins.

**Figure 5.**
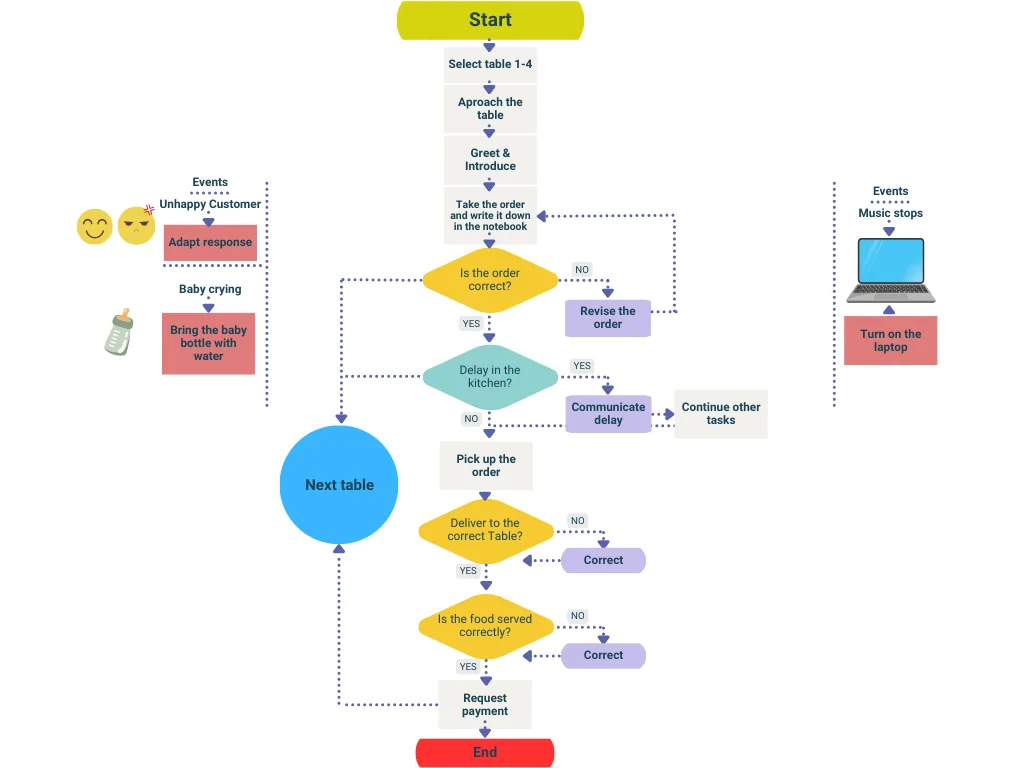
Illustration of the structure and flow of the core gameplay loop. It includes multiple simultaneous and context-sensitive interactions, such as responding to customer emotions, handling multitasking under time constraints, and managing delays or order issues, all representative of real-life executive functioning demands. This flowchart highlights how key executive domains (eg, planning, decision-making, multitasking, and contextual control) are embedded into the gameplay.

The SG is designed to integrate ecologically valid elements by simulating real-life challenges within emotionally and socially dynamic environments. As such, it provides a unique opportunity to assess EF with high contextual relevance, particularly in populations whose deficits are often undetected by conventional methods. Figure 5 shows the flow of the core gameplay loop.

The creation and design process of the SG resulted in a clinically oriented tool intended to assess the previously described EF domains in a manner that approximates real-world contexts. Table 2 shows the examples of tasks associated with each cognitive function.

**Table 2.** Cognitive skills assessed across different tasks within the serious game (SG) designed to evaluate executive dysfunction.

Cognitive function assessed	Task	SG
Contextual control	Table with 1 customer: the customer’s order is delayed and he starts to get angry. The player must choose an appropriate response to the customer’s emotional state, which can either increase his anger or calm him down.	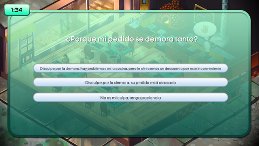
Prospective memory	Remember to play music every time it is switched off. The player is informed at the beginning that music should always be playing in the restaurant, making this a time-based prospective memory challenge.	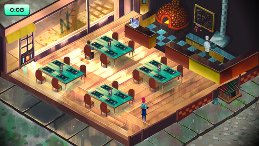
Working memory	Table with 4 customers: In total, they order 3 different meals and 3 different beverages, with repetition of one meal and one beverage (working memory load: 6 items).	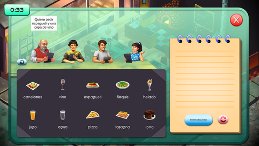
Cognitive flexibility	Table with 4 customers: one of the customers, after ordering and at the moment the dish is delivered, decides to change his order. The player is required to adapt to the change and select an appropriate course of action.	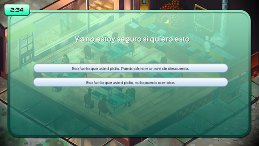
Planning	All actions or tasks performed by the assessed participant to achieve a goal. The trajectory followed by the subject—from the kitchen to each table—is evaluated to assess planning efficiency.	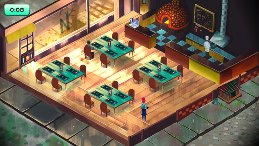
Decision-making	All situations in which the person being assessed has to choose between 2 or more actions, which will have different consequences (positive, neutral, or negative).	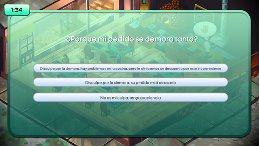
Social cognition	Ability to respond in a conventional manner to social norms. Each time the waiter starts interacting with a new customer, he/she will have the option to introduce him/herself or skip this step and take the order directly. Additional dilemmas include whether or not to return found money from one of the tables.	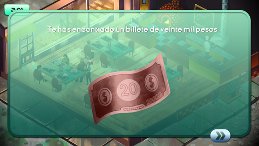
Multitasking	Simultaneous performance of multiple tasks during gameplay, including distractions such as a crying baby.	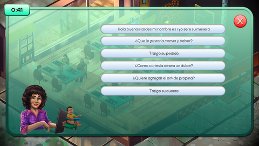

### Usability Study

#### Study Design

This study follows a cross-sectional observational design, with clinical and nonclinical participants, aimed at evaluating the usability of a clinically oriented SG developed to assess ED in ecologically valid contexts.

#### Setting

The study was conducted in 3 clinical settings in Santiago, Chile (Hospital del Salvador, Hospital Clínico de la Universidad de Chile, and Mutual de Seguridad). Data collection took place between August 2023 and February 2025, including participant recruitment, neuropsychological evaluation, and SG administration. All assessments were performed in controlled environments under the supervision of trained health professionals.

#### Sampling Procedures

**Figure 6.**
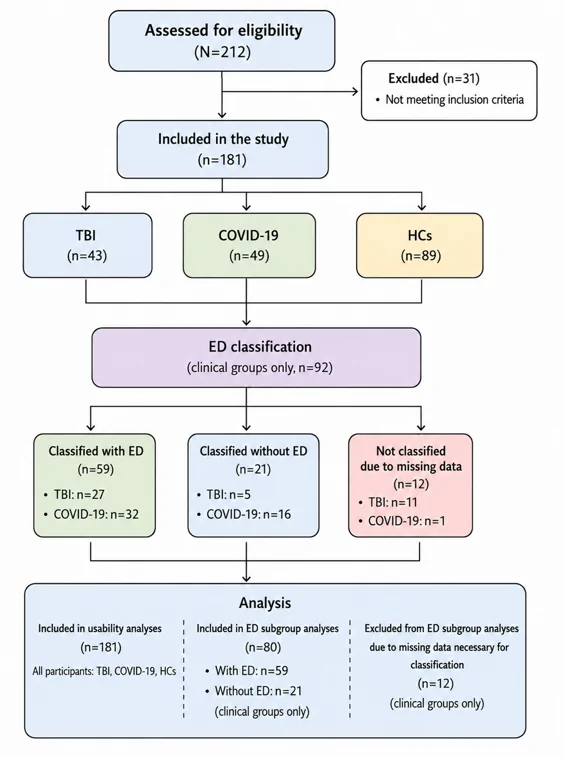
Participant flow diagram of the study. The diagram illustrates recruitment, group allocation (traumatic brain injury [TBI], COVID-19, and healthy controls [HCs]), executive dysfunction (ED) classification within clinical groups, and inclusion in the analyses. A total of 181 participants were included (43 with TBI, 49 with COVID-19, and 89 HCs). ED classification was conducted in clinical groups only (n=92), with 12 participants excluded from subgroup analyses due to missing data.

Participants were recruited through convenience sampling. Clinical participants were identified principally via referrals from health care professionals in collaborating institutions, while healthy controls (HCs) were recruited through community outreach and institutional participant registries. Initial eligibility screening was conducted via telephone, followed by in-person confirmation (Figure 6).

No monetary compensation was provided. Participants were offered a snack during a break in the evaluation session and received feedback on their neuropsychological assessment scores. It was clearly explained that these results represented psychometric findings only and did not constitute a formal clinical report.

### Participant Characteristics

#### Inclusion and Exclusion Criteria

The sample included individuals aged 21 to 66 years, comprising both clinical populations (TBI and post–COVID-19) and HCs. Eligibility criteria were defined as follows:

Participants were required to (1) have completed at least 6 years of education, (2) be able to agree to participate in the study and sign the informed consent form, and (3) be able to perform cognitive tasks for at least 20 minutes. People were not recruited if they had severe cognitive impairment (assessed via neuropsychological testing), sensory impairments not compensated by assistive technology, motor impairment of the dominant hand, speech impairment interfering with assessment, or disabling systemic disease preventing a definitive diagnosis of ED.

#### Clinical Group

Inclusion criteria included (1) COVID-19 infection requiring hospitalization (at least 3 mo ago) with self-report of at least one neurological sign (COVID group) or TBI that has required emergency department consultation or hospitalization after the TBI (TBI group) and (2) clinical history suggestive of ED according to the Dysexecutive Questionnaire [37] or reported by the clinical staff (not explained by another cause such as perceptual, motor, or other cognitive disorders). Exclusion criteria included a history of premorbid intellectual disability that has limited access to formal education.

#### HC Group

Inclusion criteria included (1) being a resident of the community and able to live independently, (2) performance in normal ranges in the Frontal Assessment Battery (Chilean version) [38], and (3) clinical consensus following neuropsychological evaluation, excluding the presence of ED. Exclusion criteria included (1) a history of major neurological and/or psychiatric disease, alcohol, or drug abuse, (2) the current use of certain psychotropic drugs, or (3) current depressive or anxiety disorder.

### Measures and Covariates

Demographic variables, including age, sex, and years of education, were recorded and included as covariates in the statistical analyses. All participants were evaluated by a clinical neuropsychologist who collected qualitative clinical data.

Neuropsychological measures included assessments of general cognitive functioning using the Montreal Cognitive Assessment [39]; attention and working memory using the Digit Span test (forward and backward) [40] and the d2-R Test of attention [41]; processing speed using the Trail Making Test Part A; and executive functioning using the Trail Making Test Part B [42], Frontal Assessment Battery [38], Tower of London Test [43], and the Dysexecutive Questionnaire [37] administrated to the participant and a reliable proxy. Finally, daily functioning abilities were evaluated using the Technology–Activities of Daily Living Questionnaire [44], administered both to the participant and the proxy.

The primary outcomes of interest were usability and perceived difficulty, assessed using the System Usability Scale (SUS) [45] and the Perceived Difficulty Scale (DP13-CL) [46], respectively.

SUS: The SUS is one of the most widely used questionnaires to test usability [32]. It evaluates the effectiveness, efficiency, and satisfaction of participants’ interaction with a system by means of 10 items answered with a Likert scale ranging from 1 (“strongly disagree”) to 5 (“strongly agree”). Sample items include: *I think I would use this system frequently*, *I find this system unnecessarily complex*, and *I think the system was easy to use*, among others.
DP13-CL (Adaptation of the DP-15 Scale): This scale was specifically designed to assess perceived difficulty in the SG. Respondents rate their difficulties on a numerical scale, where level 1 denotes “no difficulty” (“extremely easy”) and level 13 represents “maximum difficulty” (“extremely difficult”).

### ED Classification

Clinical consensus regarding ED was established through multidisciplinary case discussions involving neurologists and neuropsychologists, after the completion of each participant’s neuropsychological evaluation. ED was operationally defined based on a combination of standardized neuropsychological test performance and expert clinical consensus, which served as the reference standard for group classification.

Participants were classified into 3 categories: no ED, mild ED, or moderate to severe ED, based on the integration of standardized test performance, deviations from normative values, and functional impact. In cases of disagreement, consensus was reached through discussion among clinicians. For the purposes of the present analyses, participants classified as mild or moderate to severe were grouped as presenting ED.

Importantly, ED classification was independent of the clinical group, such that participants within the TBI and COVID-19 groups could be classified as with ED (27 participants with TBI and 32 participants with COVID-19) or without ED (5 participants with TBI and 16 with COVID-19) based on the consensus procedure.

It is worth noting that 12 out of 92 participants within the clinical groups (11 with TBI and 1 with COVID-19), representing approximately 13% of the clinical sample, could not be classified regarding the presence or absence of ED due to missing clinical or informant data required for consensus-based classification. Consequently, these participants were excluded from analyses that required ED classification. No imputation procedures were applied, as the missing data affected only the classification variable and not the primary usability outcomes.

### Sample Size, Power, and Precision

The study sample size (N=181) was determined based on feasibility considerations, including participant availability within the recruitment period and resources for comprehensive neuropsychological assessment. While no formal a priori sample size calculation was conducted given the primary focus on usability evaluation rather than hypothesis testing, a post hoc power analysis confirmed the robustness of our comparative findings. Specifically, for our main contrast regarding perceived usability between clinical patients with ED (n=59) and HC (n=89), the analysis indicated an adequate effect size (Cohen *d*=0.588), yielding a statistical power of 93.5% (1–β=.935) to detect meaningful differences in SUS scores at an α level of .05. Furthermore, to address estimation precision, 95% CIs were computed and reported for all key usability metrics and point estimates.

### Statistical Analysis

Descriptive statistics were computed for all variables. Group differences in continuous variables were assessed using nonparametric tests (Kruskal-Wallis and Mann-Whitney tests), while categorical variables were analyzed using chi-square tests. To indicate the precision of our estimates, 95% CIs were calculated for the main group comparisons. Sociodemographic variables were used as covariates when corresponding.

To control for potential confounders, linear models were used to analyze SUS scores, including group and sex as fixed factors and age and education as covariates. Ordinal logistic regression models were applied to analyze perceived difficulty (DP13-CL), using the same covariates.

All statistical analyses were conducted using Jamovi software (The Jamovi Project), with statistical significance set at *P*<.05.

### Ethical Considerations

This study was approved by the Ethics Committee of Hospital Clínico de la Universidad de Chile (approval number OAIC 1300/22), covering research activities conducted at Hospital Clínico de la Universidad de Chile and Hospital del Salvador, and by the Ethics Committee of Mutual de Seguridad (approval number 20CV-140571). The study was conducted in accordance with the ethical principles of the Declaration of Helsinki and applicable national regulations governing research involving human participants. Participants were recruited and assessed at Hospital Clínico de la Universidad de Chile, Hospital del Salvador, and Mutual de Seguridad. Written informed consent was obtained from all participants prior to study participation. No financial compensation was provided. However, participants were offered a light refreshment during the assessment session and received individualized feedback on their neuropsychological assessment results.

All data were anonymized prior to analysis to ensure participant confidentiality, using coded identifiers. Data were securely stored using REDCap electronic data capture tools hosted at the Faculty of Medicine, Universidad de Chile [47], and were accessible only to authorized members of the research team. No identifiable participant data or images are included in this paper or supplementary materials.

## Results

### Perceived Usability and Difficulty

A total of 212 participants were assessed for eligibility in the usability study, of whom 181 were included in the final analyses. The final sample consisted of 43 participants with TBI, 49 participants with COVID-19, and 89 HCs. Table 3 presents the demographic characteristics of the sample, including age, years of education, sex distribution, and the mean scores on the neuropsychological tests and questionnaires administered during the assessment.

**Figure 7.**
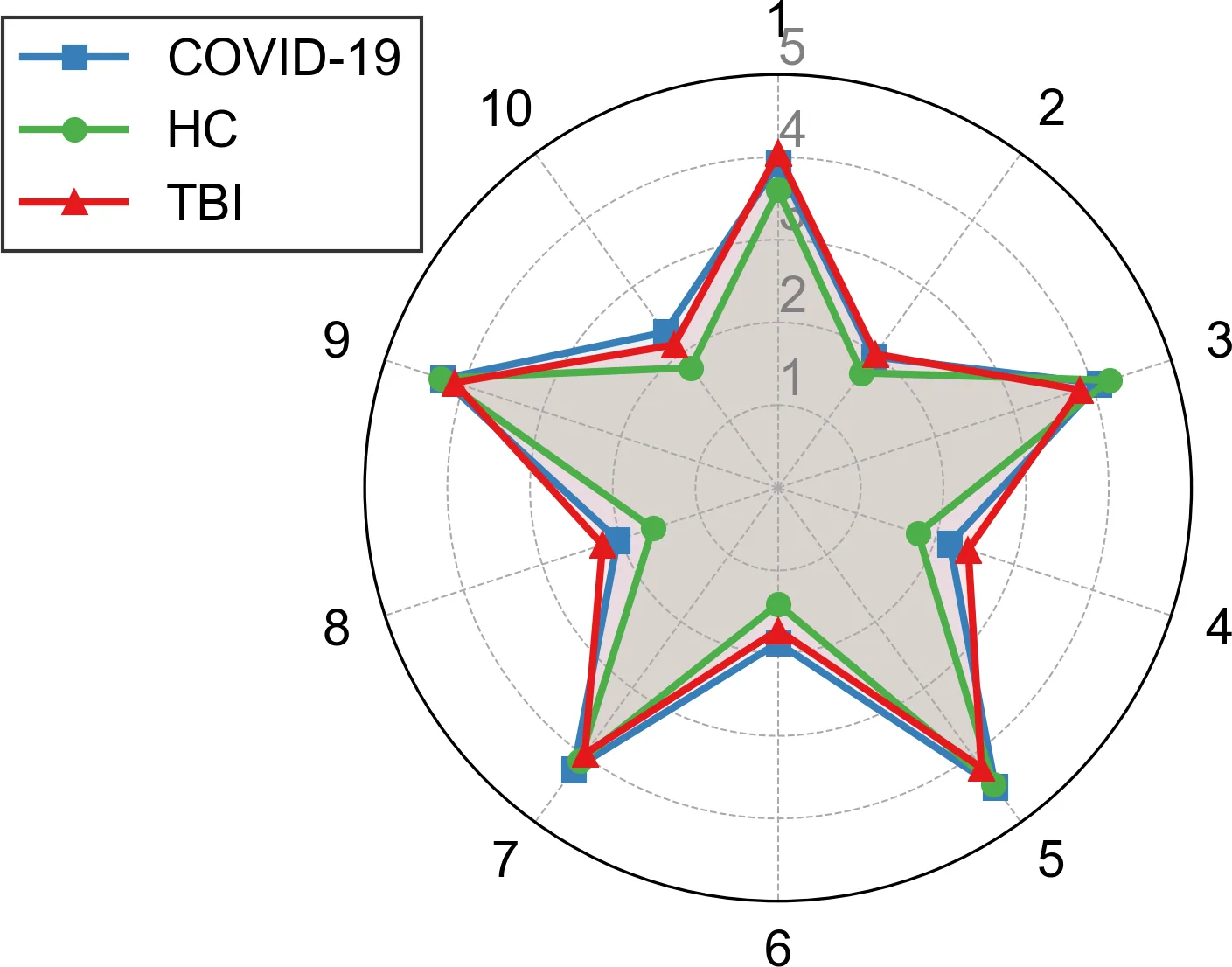
Spider plot representing the mean scores for each item of the System Usability Scale (SUS) across groups. The plot displays the mean score (range 1‐5) for each of the 10 SUS items: (1) “I think that I would like to use this system frequently,” (2) “I found the system unnecessarily complex,” (3) “I thought the system was easy to use,” (4) “I think that I would need the support of a technical person to be able to use this system,” (5) “I found the various functions in this system were well integrated,” (6) “I thought there was too much inconsistency in this system,” (7) “I would imagine that most people would learn to use this system very quickly,” (8) “I found the system very cumbersome to use,” (9) “I felt very confident using the system,” (10) “I needed to learn a lot of things before I could get going with this system.” HC: healthy control; TBI: traumatic brain injury.

The analysis of total scores on the SUS and DP13-CL is presented in Table 3. The item-level breakdown of the SUS is shown in Figure 7. According to the classification proposed by Bangor et al [48], SUS scores indicated good usability across all groups. A Kruskal-Wallis test revealed significant differences between groups (*χ*²_2_=9.18, *P*=.01). Post hoc pairwise comparisons showed that the TBI group rated usability significantly lower than the HC group (*P*=.02). No significant differences were found between the TBI and COVID groups (*P*=.93) or between the COVID and HC groups (*P*=.06).

For the DP13-CL, the overall mean score across all participants was 5.61 (SD 2.36), corresponding to an average difficulty rating between “easy” and “neither easy nor difficult.” Reported scores ranged from 1 (“extremely easy”) to 12 (“very difficult” to “extremely difficult”), with a total sample size of 181. Group-level analysis revealed that the COVID group reported the highest perceived difficulty (mean 6.43, SD 2.29), followed by the TBI group (mean 5.79, SD 2.61) and the HC group (mean 5.08, SD 2.14). A Kruskal-Wallis test indicated significant differences among the 3 groups (*χ*²_2_=11.6, *P*=.003). Post hoc pairwise comparisons showed a significant difference between the COVID and HC groups (*P*=.001), while no significant differences were observed between the TBI and COVID groups (*P*=.63) or between the TBI and HC groups (*P*=.23).

**Table 3.** Demographic characteristics of the clinical group and healthy controls (HC)^a^.

Characteristic	TBI^b^ (n=43), mean (SD; range)	COVID (n=49), mean (SD; range)	HC (n=89), mean (SD; range)	Median (IQR)	*P* value
Age	48.4 (11.0)	48.7 (10.9)	42.9 (13.6)	48.0 (21.0-66.0)	.04
Education	12.3 (3.3)	14.3 (3.1)	17.2 (2.8)	16.0 (6.00-26.0)	<.001
Sex (male/female)	24/18	18/31	25/64	—^c^	.006
MoCA^d^	24.0 (4.6)	24.4 (3.8)	27.2 (2.2)	26.0 (13.0-30.0)	<.001
FAB^e^	14.5 (2.6)	15.3 (2.4)	17.0 (1.0)	16.0 (7.00-18.0)	<.001
GREFEX^f^	1.97 (1.56)	1.59 (1.89)	0.25 (0.53)	0 (0-7)	<.001
SUS^g^	73.9 (17.6; 68.5‐79.3)	76.5 (14.5; 72.3‐80.7)	81.7 (13.5; 78.9‐84.5)	80.0 (12.5-100)	.01
DP13-CL^h^	5.79 (2.61; 5.0‐6.6)	6.43 (2.29; 5.8‐7.1)	5.08 (2.14; 4.6‐5.5)	6.00 (1-12)	.003

a Continuous variables were compared using the Kruskal-Wallis test, and sex was compared using the chi-square test. MoCA and FAB scores are reported to reflect general and frontal cognitive functioning, respectively. GREFEX indicates symptom reports related to the behavioral dysexecutive syndrome, with the presence of three or more symptoms suggesting the presence of this syndrome. Seven participants from the TBI group and 2 from the HC group did not have GREFEX data due to difficulties coordinating interviews with their respective informants.

b TBI: traumatic brain injury.

c Not applicable.

d MoCA: Montreal Cognitive Assessment.

e FAB: Frontal Assessment Battery.

f GREFEX: Groupe de Réflexion sur l'Évaluation des Fonctions Exécutives.

g SUS*: *System Usability Scale.

h DP13-CL: Perceived Difficulty Scale.

When analyses were restricted to participants with ED from the COVID and TBI groups (n=59), they exhibited significantly lower SUS scores (mean 73.1, SD 16.2; *P*<.001) and higher perceived difficulty (mean 6.34, SD 2.32), compared to HCs (SUS: mean 81.7, SD 13.5; DP13-CL: mean 5.08, SD 2.14; Table 4).

Given the observed group differences in age and education, additional analyses were conducted to control for these demographic variables. A general linear model was used to analyze SUS scores, with group and sex as fixed factors and age and years of education entered as covariates. The model was significant (*F*_7,171_=4.32, *P*<.001, *R*²=0.150). However, no significant main effects of group (*P*=.51) or sex (*P*=.86) were observed, and the group × sex interaction was not significant (*P*=.28). Notably, age was negatively associated with SUS scores (β=–.272, *P*=.003), while education was positively associated (β=.794, *P*=.04). These findings suggest that the initial group differences observed in unadjusted analyses were largely explained by individual differences in age and education.

To explore whether the same pattern was held for perceived difficulty (DP13-CL), an ordinal logistic regression (proportional odds model) was conducted using group and sex as fixed factors, with age and education as covariates. The model was significant (*χ*²_7_=34.4, *P*<.001, *R*²=0.044). As in the SUS analysis, group (*P*=.08), sex (*P*=.59), and the group × sex interaction (*P*=.31) were not significant. However, age was positively associated with perceived difficulty (odds ratio 1.04, 95% CI 1.02-1.07, *P*<.001), indicating that older participants reported higher difficulty. Years of education showed a trend-level negative association with perceived difficulty (odds ratio 0.91, 95% CI .83-1.00, *P*=.05), suggesting that higher educational attainment was associated with lower perceived difficulty.

**Table 4.** System Usability Scale (SUS) and Perceived Difficulty Scale (DP13-CL) scores across groups^a^.

Type of scale	ED^b^ (n=59), mean (SD; 95% CI)	HC^c^ (n=89), mean (SD; 95% CI)	*P* value
SUS	73.1 (16.2; 68.9‐77.3)	81.7 (13.5; 78.8‐84.5)	<.001
DP13-CL	6.34 (2.32; 5.73‐6.94)	5.08 (2.14; 4.63‐5.53)	<.001

a Mann-Whitney different means with *P*<.001.

b ED: executive dysfunction.

c HC: healthy control.

## Discussion

### Principal Findings

The present study had two aims: (1) to describe the development of a clinically oriented SG designed to assess ED in ecologically valid contexts and (2) to evaluate its usability in clinical and healthy populations. Overall, usability was rated as good across groups, and perceived difficulty fell within a moderate range. Participants with ED reported lower usability and higher perceived difficulty compared to HCs, although these differences were attenuated after adjusting for age and education. Age and educational level were significantly associated with usability and perceived difficulty, indicating that user characteristics influence interaction with the tool.

### Interpretation

Differences in perceived usability and difficulty in older and less educated participants are consistent with prior findings indicating that age affects usability perceptions, in which task difficulty may be over- or underestimated regardless of objective performance [49]. Regarding education, it is noteworthy that participants with lower educational backgrounds were mainly from the TBI group, which also showed more pronounced ED. Previous studies have documented the relationship between lower education and greater executive deficits in TBI [50], suggesting that usability differences may partly reflect underlying executive impairment, while also highlighting the role of demographic factors in shaping user experience [49]. The relatively narrow confidence intervals observed for these estimates suggest an acceptable level of precision [51], although some variability remains, particularly in subgroup analyses. Future studies are needed to determine the extent to which demographic factors influence the diagnostic accuracy of the SG.

As highlighted by Martínez-Pernía et al [14], traditional tools lack ecological validity, whereas SGs can more accurately simulate the demands of everyday life. A key advantage of the current SG lies in its ability to recreate realistic, dynamic, and socially embedded situations relevant to everyday functioning. Participants with ED, across both the post–COVID-19 and TBI groups, showed lower usability ratings and higher perceived difficulty compared to HCs. These group differences are consistent with previous literature, suggesting that individuals with ED experience greater difficulties when interacting with complex and dynamic environments [14]. Such contexts often involve emotionally salient and embodied interactions (ie, the integration of cognitive, emotional, physiological, and bodily responses during real-world social interactions) [14,52,53], which are crucial for effective executive functioning in daily life. However, these features may also increase task complexity and cognitive load [22]. Importantly, ecological validity does not necessarily imply lower task demands or greater usability; rather, more realistic scenarios may inherently increase cognitive load [14,22,23]. Therefore, the observed differences may reflect both the sensitivity of the task to executive impairments and the increased complexity associated with these context-rich environments, and its utility for diagnostic accuracy remains to be established. These findings should be interpreted in light of the adjusted analyses, in which group differences were attenuated after controlling for age and education. Accordingly, the results are better understood as reflecting the interaction between user characteristics and task demands within realistic assessment contexts, rather than as evidence of differential sensitivity to ED.

Unlike earlier usability studies that relied exclusively on healthy participants (eg, [16,27,28]), the inclusion of clinical groups in this study provides important evidence for the feasibility and acceptability of the SG in its intended population, thereby enhancing its translational relevance. Although usability testing can yield meaningful insights with relatively small samples, larger sample sizes increase confidence in the identification of usability issues. As Faulkner [54] noted, “more test users means greater confidence that the problems that need to be fixed will be found” (p. 382).

### Implications

SGs have emerged as promising tools for cognitive assessment, with the potential to address limitations of traditional neuropsychological measures by providing more engaging, dynamic, and ecologically valid contexts [14,18,19,21]. The present findings support the feasibility of this approach in clinical populations, demonstrating that a complex, ecologically valid SG can be used with acceptable usability across diverse groups. Importantly, the influence of age and education on usability highlights the need to consider demographic factors in the design, interpretation, and implementation of SG-based assessments. Future studies should explore the development of adjusted norms, stratification strategies, or interface adaptations to ensure accessibility and appropriate interpretation across populations [18,55].

Although the present study focused on development and usability rather than diagnostic validity, the platform offers a promising foundation for future validation work [14,18,19]. Subsequent research should evaluate its sensitivity and specificity by comparing gameplay-derived performance with gold-standard neuropsychological measures and clinical consensus classifications of ED. Given its digital and interactive nature, the SG also has potential for broader applications, including screening, longitudinal monitoring, and integration into stepped-care models [56]. However, its use was evaluated in a supervised clinical context, and further research is needed to establish its feasibility, reliability, and interpretability in unsupervised or real-world settings.

### Limitations

This study has several limitations that should be considered when interpreting the findings. First, usability was assessed using self-reported measures, which may be subject to response biases and do not fully capture actual user behavior or task engagement [57,58]. Although the platform records detailed behavioral data (eg, action sequences, timing, and errors), these were not analyzed in the present study, which focused on development and initial usability, and therefore represent an important direction for future research. Second, the study did not evaluate the diagnostic validity of the SG; therefore, no conclusions can be drawn regarding its ability to detect ED or differentiate between clinical conditions. Third, the observed influence of age and education highlights potential variability in user experience, which may limit the generalizability of the findings across populations with different demographic characteristics. Finally, the SG was evaluated within a supervised clinical context, and its feasibility, reliability, and user interaction patterns in unsupervised or real-world settings remain to be established and should be examined in future studies. Nonetheless, a major strength of this study is the development of a novel SG for the assessment of ED, as well as the reporting of usability and perceived difficulty data in both healthy participants and individuals with ED from different etiologies.

### Conclusion

The SG showed good usability and represents a promising platform for future validation studies. Its design supports the development of more contextually grounded approaches to the assessment of executive functioning and provides the initial evidence of feasibility in clinical populations. These findings contribute to the growing use of interactive, context-rich digital tools in neuropsychological assessment, addressing longstanding limitations of traditional methods in capturing everyday cognitive functioning. As digital health technologies continue to evolve, ensuring usability and accessibility will be critical for successful clinical implementation. Future research should incorporate objective behavioral metrics derived from gameplay, alongside self-report measures, to achieve a more comprehensive characterization of user interaction and performance. Further work is also needed to establish diagnostic accuracy and clinical reliability and to integrate such tools into routine neuropsychological assessment.

## Abbreviations

DP13-CL: Perceived Difficulty Scale
ED: executive dysfunction
EF: executive function
SG: serious game
SUS: System Usability Scale
TBI: traumatic brain injury
